# Overcoming translational barriers in H3K27-altered diffuse midline glioma: Increasing the drug-tumor residence time

**DOI:** 10.1093/noajnl/vdad033

**Published:** 2023-03-27

**Authors:** Erica A Power, Julian S Rechberger, Liang Zhang, Ju-Hee Oh, Jacob B Anderson, Cody L Nesvick, Jizhi Ge, Edward H Hinchcliffe, William F Elmquist, David J Daniels

**Affiliations:** Department of Neurologic Surgery, Mayo Clinic, Rochester, Minnesota, USA; Department of Neurologic Surgery, Mayo Clinic, Rochester, Minnesota, USA; Department of Molecular Pharmacology and Experimental Therapeutics, Mayo Clinic, Rochester, Minnesota, USA; Department of Neurologic Surgery, Mayo Clinic, Rochester, Minnesota, USA; Brain Barriers Research Center, Department of Pharmaceutics, College of Pharmacy, University of Minnesota, Minneapolis, Minnesota, USA; Department of Neurologic Surgery, Mayo Clinic, Rochester, Minnesota, USA; Department of Molecular Pharmacology and Experimental Therapeutics, Mayo Clinic, Rochester, Minnesota, USA; Department of Neurologic Surgery, Mayo Clinic, Rochester, Minnesota, USA; Department of Neurologic Surgery, Mayo Clinic, Rochester, Minnesota, USA; Hormel Institute, University of Minnesota, Austin, Minnesota, USA; Brain Barriers Research Center, Department of Pharmaceutics, College of Pharmacy, University of Minnesota, Minneapolis, Minnesota, USA; Department of Neurologic Surgery, Mayo Clinic, Rochester, Minnesota, USA; Department of Molecular Pharmacology and Experimental Therapeutics, Mayo Clinic, Rochester, Minnesota, USA

**Keywords:** aurora kinase, alisertib, convection-enhanced delivery, drug-tumor residence time, Diffuse midline glioma

## Abstract

**Background:**

H3K27-altered diffuse midline glioma (DMG) is the deadliest pediatric brain tumor; despite intensive research efforts, every clinical trial to date has failed. Is this because we are choosing the wrong drugs? Or are drug delivery and other pharmacokinetic variables at play? We hypothesize that the answer is likely a combination, where optimization may result in a much needed novel therapeutic approach.

**Methods:**

We used in vitro drug screening, patient samples, and shRNA knockdown models to identify an upregulated target in DMG. A single small molecule protein kinase inhibitor with translational potential was selected for systemic and direct, loco-regional delivery to patient-derived xenografts (PDX) and genetically engineered mouse models (GEMM). Pharmacokinetic studies were conducted in non-tumor bearing rats.

**Results:**

Aurora kinase (AK) inhibitors demonstrated strong antitumor effects in DMG drug screens. Additional in vitro studies corroborated the importance of AK to DMG survival. Systemic delivery of alisertib showed promise in subcutaneous PDX but not intracranial GEMM and PDX models. Repeated loco-regional drug administration into the tumor through convection-enhanced delivery (CED) was equally inefficacious, and pharmacokinetic studies revealed rapid clearance of alisertib from the brain. In an effort to increase the drug to tumor residence time, continuous CED over 7 days improved drug retention in the rodent brainstem and significantly extended survival in both orthotopic PDXs and GEMMs.

**Conclusions:**

These studies provide evidence for increasing drug-tumor residence time of promising targeted therapies via extended CED as a valuable treatment strategy for DMG.

**Key Points**

- Aurora kinase is a promising target in H3K27-altered DMG.
- The aurora kinase inhibitor alisertib is rapidly cleared from brain following direct delivery.
- Prolonged convection-enhanced delivery of alisertib improves survival in orthotopic DMG animal models.

**Importance of the Study**

The most aggressive and lethal of all pediatric brain tumors are H3K27-altered diffuse midline gliomas (DMG). Over 100 clinical trials have failed to show any therapeutic benefit, and our failures with this tumor are likely multifactorial. We identified and demonstrated the importance of aurora kinases (AK) to DMG tumorigenesis. While oral treatment of subcutaneous DMG xenograft with the AK inhibitor alisertib reduced tumor size, this result was not replicated in multiple intracranial models, nor did it show success following repeated loco-regional administration via convection-enhanced delivery (CED). After optimization of drug to tumor-residence time using an extended CED regimen to deliver alisertib for 7 days, we show increased survival in both genetically engineered mouse models (GEMM) and patient-derived xenografts (PDX) models. Our findings highlight the significance of increasing the drug-tumor residence time as a translational pathway that may ultimately find success in treating this devastating disease.

Diffuse midline glioma (DMG) harboring Lysine residue at position 27 (K27) of histone subunit 3 (H3) (H3K27)alterations, previously known as diffuse intrinsic pontine glioma when located in the brainstem, is an aggressive and uniformly lethal central nervous system (CNS) cancer that predominately occurs in children but occasionally adults.^1^ The prognosis for this disease is dismal with a median survival of less than 1 year following diagnosis. Despite decades of research, clinical trials have yet to find an effective therapy.^2^ The current standard of care includes radiation therapy, which is largely palliative, alleviating some symptoms for a short period of time without providing a significant improvement in overall survival.^3^ There is an urgent unmet need to offer novel treatment strategies and give hope to afflicted patients and their families.

Landmark studies in 2012 revealed that almost all DMGs contain a missense mutation in *H3F3A* or *HISTH13B*, resulting in a lysine-to-methionine substitution at position 27 on histone H3 (H3K27M).^4,5^ Since then, research aimed at understanding molecular drivers of this disease has flourished. The H3K27M mutation results in a global reduction of H3K27 trimethylation (H3K27me3) on wild-type histone proteins and consequently, a reprogramming of the cell’s epigenetic landscape.^6^ These changes are often accompanied by additional oncogenic mutations such as loss of p53 and mutated Platelet-derived growth factor receptor α (PDGFRα).^7–9^ It is thought that these combined mutations promote a more stem-like phenotype and drive tumorigenesis.^10^ However, despite rigorous mechanistic studies, there has been no translation into a clinically efficacious treatment strategy.

The purpose of this study was to identify targeted agents for DMG and assess the translational potential as a novel and efficacious treatment approach. We screened a library of epigenetic regulators in multiple H3K27M and H3K27-WT patient-derived cell lines and identified that aurora kinase (AK) inhibitors have strong and somewhat selective antitumor effects. We further demonstrated the importance of AK to H3K27M DMG growth and proliferation. Alisertib was chosen as the lead compound, as it has an established safety profile in children. When we administered alisertib orally in a H3K27M genetically engineered mouse model (GEMM), we did not see a survival benefit. Surprisingly, even after direct administration by convection-enhanced delivery (CED), we did not find efficacy with alisertib. Our pharmacokinetic (PK) studies subsequently revealed that alisertib is rapidly cleared from the brain following acute CED. For this reasons, we decided to administer alisertib directly to the tumor over an extended period of time (7 days) and finally found a survival benefit and on-target drug effects in both GEMM and patient-derived xenograft (PDX) models. These data coupled with other reports highlight the importance of drug-tumor residence as being a key contributor to the development and translation of novel therapies for DMG.

## Materials and Methods

### Cell Lines and Culture

Institutional Review Board approval and informed consent were obtained for all human tissue studies and patient-derived cell lines. Detailed information regarding cell line origin, molecular status, and other pertinent data including media for each cell line can be found in Supplementary Tables 1 and 2. Cells were passaged every 1–2 weeks for neurospheres and 1–2 times per week for adherent monolayers.

### Western Blot

Protein lysates from patient samples were obtained from the Mayo Clinic and the DIPG Registry and Repository (Cincinnati Children’s Hospital Medical Center). In all cases, proper Institutional Review Board approval and consent were obtained. Standard Western blot techniques were used as previously described and are detailed in (Supplementary Methods, Table S3).^11^

### RNA Extraction and Next Generation Sequencing

A RNeasy Plus micro kit (QIAGEN) was used to extract whole RNA per the manufacturer’s instructions. RNA quality, library preparation, and sequencing were performed by Novogene. RNA sample integrity, purity, and quantitation were validated using NanoPhotometer spectrophotometer (IMPLEN), 1% agarose gel electrophoresis, and Bioanalyzer 2100 (Agilent Technologies). RNA sample preparation was done using 1 μg of RNA per sample and sequencing libraries were generated with a NEBNext UltraTM RNA Library Prep Kit from Illumina (New England Biolabs) per the manufacturer’s instructions. Then, cDNA fragments 150-200bp in length were selected using AMPure XP magnetic beads (Beckman Coulter). Final library quality was confirmed with an Agilent Bioanalyzer 2100. An Illumina Novaseq 6000 sequencer was used to perform clustering of the index-coded samples per the manufacturer’s instructions. Finally, after cluster generation, libraries were sequenced on the same machine and paired-end reads were generated. Detailed methods describing RNA-seq analysis can be found in Supplementary Methods.

### Inducible AURKA/AURKB Knockdown in DMG Cells

DMG cell lines (SF8628 and DIPGXVII) were transduced with a doxycycline (DOX)-inducible AURKA and AURKB shRNA (iSMART inducible dox system, Horizon Discovery). Positively transduced cells were selected with puromycin (1μg/mL, Gibco) for 5 days.

### Cell Proliferation Assay

Using a 96-well plate (Corning), cells were seeded at a density of 3 × 10^4^ cells per well and were incubated overnight. The next morning, the 96-well plates were placed into the IncuCyte S3 (Essen Bioscience) and imaged every 3 hours. Cell confluency was obtained via Incucyte S3 2018A software (Essen Bioscience).

### Animal Studies

All animal procedures were approved by the Mayo Clinic Institutional Animal Care and Use Committee. Human tissue samples from which xenografts are derived were obtained with approval from the necessary Institutional Review Board (Stanford University, Mayo Clinic) and the patients are since deceased. All animal survival surgeries were conducted under aseptic conditions using 2% isoflurane inhalation as an anesthetic. Details regarding each animal study and details pertaining to imaging modalities used in animal studies can be found in Supplementary Methods.

### Immunohistochemistry

Animals were euthanized and the brains were removed and fixed in 4% paraformaldehyde. Then, the brains were embedded in paraffin prior to sectioning with a microtome (5 μM tissue sections). Hematoxylin and eosin (H&E) stains and immunohistochemistry (IHC) stains were done per standard procedures, and details regarding the IHC staining are described in (Supplementary Methods, Table S3).

### Plasma and Brain Drug Concentration by LC-MS/MS

Alisertib concentration in the brain and plasma was determined by LC-MS/MS after a liquid–liquid extraction, details of which can be found in Supplementary Methods.

### Data Availability

Some data were obtained from R2: Genomics Analysis and Visualization Platform at r2.amc.nl. Additional data generated in this study are available within the article and supplementary files while raw data and derived data from RNA-seq are available upon reasonable request from the corresponding author.

## Results

### AK as a Druggable Target in H3K27M DMG

**Figure 1.**
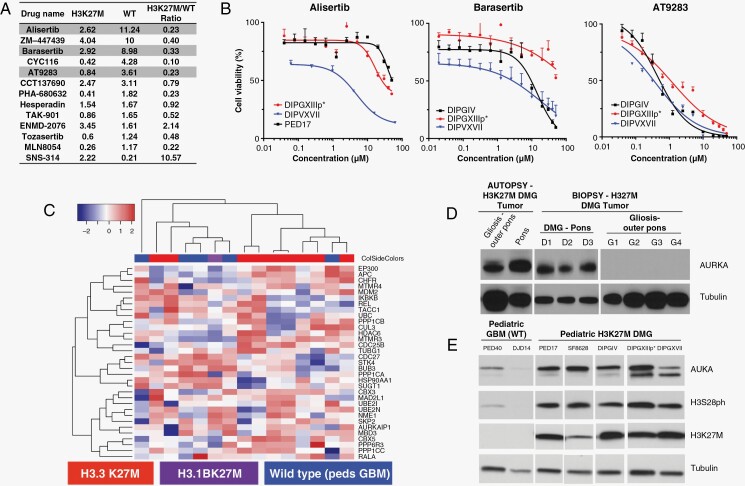
Epigenetic drug screen identifies aurora kinase (AK) as a druggable target and is upregulated in diffuse midline glioma (DMG) with the H3K27M mutation. (A) IC_50_ values (μM) and K27M/WT IC_50_ ratio for AK inhibitors (AKIs) from in vitro drug screen of clinically relevant epigenetic regulators (*n* = 359). Each drug was tested in triplicate with 2–3 independent experiments (*n* = 6–9) in each cell line. (B) Cell viability curves for 3 AKIs (alisertib, barasertib, AT9283) that have been or are in clinical testing with strong K27M/WT IC_50_ ratios. (C) Heatmap of RNA-Seq normalized reads of known genes associated with AK signaling. (D) Western blot of H3K27M DMG samples from noted anatomical regions, as indicated. (E) Western blot of H3K27M and H3-WT patient-derived cell lines, as indicated.

To identify novel therapeutic targets in H3K27M-mutated DMG, we performed cell viability drug screening of 359 epigenetic regulators (Supplementary Figure S1). We screened a minimum of 3 H3K27M DMG (PED8, PED17, SF8628, SU-DIPGXIII, SU-DIPGXIIIp*, and SU-DIPGXVII) and at least one H3K27-WT high-grade glioma (SF9427 and/or BT114) patient-derived cell line. Among the top results were several previously identified classes of compounds such as histone deacetylase inhibitors, including panobinostat,^12^ and K27 demethylase () inhibitors.^13^ As a class of drugs, aurora kinase inhibitors (AKIs) demonstrated potent, JMJD3H3K27M-targeted antitumor effects (Figure 1A, Supplementary Figure S1). Three AKIs that have recently been or are currently in clinical testing, alisertib (AURKA inhibitor), barasertib (AURKB inhibitor), and AT9283 (pan AK A/B/C inhibitor), preferentially decreased cell viability and proliferation of multiple H3K27M DMG lines (Figure 1B).

### AK is Differentially Expressed in H3K27M DMG

To further address AK as a potential therapeutic target for H3K27M DMG, we first accessed publicly available clinical datasets from R2: Genomics Analysis and Visualization Platform (r2.amc.nl). Diffuse intrinsic pontine glioma (DIPG) patients had significantly higher expression of AURKA compared to normal brain controls (Supplementary Figure S2A) and survival analysis of pediatric patients with high-grade gliomas indicated that high AURKA expression was associated with a poorer prognosis (Supplementary Figure S2B). Similarly, DIPG patients had high expression of Aurora kinase B (AURKB) compared with normal brain controls (Supplementary Figure S2C), but patient survival was not impacted by AURKB expression levels (Supplementary Figure S2D). Next, we conducted RNA-Seq in H3.3K27M, H3.1K27M, and H3-WT (pediatric GBM) patient-derived cell lines and analyzed transcripts for abundance of genes known to be associated with AK signaling (Figure 1C). Hierarchical clustering analysis discriminated H3K27M and H3-WT cell lines based on expression of AK-associated genes.

We then sought to evaluate protein expression of AURKA in both patient samples and patient-derived cell lines. H3K27M DMG patient tumor samples demonstrated high AURKA protein levels while tissue samples from gliosis patients did not have any AURKA expression (Figure 1D). Similar results were observed in H3K27M patient-derived cell lines, with high amounts of AURKA protein and phosphorylated H3S28 (H3S28ph), an established target of AKs.^14^ This contrasted with low detection of AURKA and H3S28ph in H3-WT pediatric GBM cell lines (Figure 1E). Together, these data indicate that upregulation of AK at both RNA and protein levels is somewhat unique to and potentially targetable in H3K27M gliomas.

### AK is Essential for H3K27M DMG Cell Proliferation and Survival

**Figure 2.**
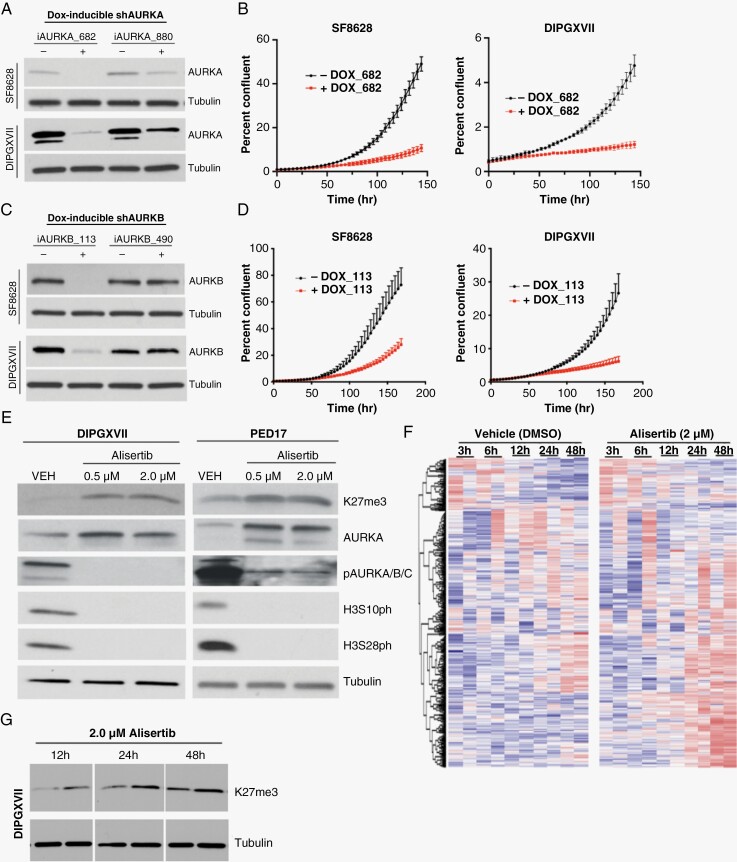
Aurora kinase (AK) is necessary for H3K27M diffuse midline glioma (DMG) cell proliferation. (A) Western blot demonstrating doxycycline (DOX)-inducible shRNA of AURKA. (B) Proliferation assay of H3K27M DMG cell lines, SF8628 and DIPGXVII, following inducible knockdown of AURKA. (C) Western blot demonstrating DOX-inducible shRNA of AURKB. (D) Proliferation assay of H3K27M cell lines, SF8628 and DIPGXVII, following inducible knockdown of AURKB. (E) Western blot showing changes in H3K27 trimethylation (H3K27me3), AURKA expression, and AURKA phosphorylation (pAURKA/B/C), as well as H3S10 and H3S28 phosphorylation (H3S10ph and H3S28ph, respectively) in DIPGXVII and PED17 cells following alisertib treatment. (F) Heatmap of RNA-Seq normalized reads of known genes associated with AK signaling following 2.0 μM alisertib treatment at indicated time points, *n* = 3 biological replicates. (G) Western blot marking changes in H3K27me3 following 2.0 μM alisertib treatment at 12 , 24, and 48 hours.

In order to elucidate the importance of AK activity to H3K27M DMG survival and proliferation, we generated DOX-inducible shRNA knockdown AURKA and AURKB models in SF8628 and DIPGXVII cell lines. Successful knockdown of AURKA was confirmed by Western blot (Figure 2A). Cell proliferation, as determined by Incucyte, was significantly lower in DOX-induced AURKA knockdown cells compared to controls in both cell lines (Figure 2B). In a similar manner, DOX-induced AURKB knockdown was confirmed by Western blot (Figure 2C), and cell proliferation was markedly decreased in the AURKB knockdown (Figure 2D).

A number of AKI compounds had a strong and somewhat selective effect on the survival of our H3K27M patient-derived cell lines. Alisertib is known to inhibit AURKA and AURKB at high concentrations (>100nM)^15^ and has been shown to be safe in children with atypical teratoid rhabdoid tumor,^16,17^ suggesting amenability for translation into the clinic in a timely manner.

DMG is partially an epigenetically driven disease. These tumors generally show low levels of H3K27me3, and there are additional changes to the histone H3 tail, including increased H3S28ph and H3S10ph and methylation changes in other important lysine residues. Pathak et al^18^ reported that as many as 66% of DMG tumors had loss of H3K4me3 as well as 44% having loss of H3K9me3. These changes lead to a reprogramming of the epigenetic landscape, resulting in global changes to gene expression and cells ultimately maintaining a more stem-like state.^10,19^ To understand the effects of alisertib on the epigenetic signature in these tumors, we treated H3K27M patient-derived cells with alisertib for 72 hours and assayed protein levels of pertinent markers. Initial assessment showed a clear increase in H3K27me3 and AURKA along with a robust decrease in activated, phosphorylated AK as well as decreased H3S28ph and H3S10ph in cells treated with alisertib (Figure 2E). We also assessed methylation changes in other lysine residues along the histone H3 tail (H3K4, H3K9, H3K20, and H3K36), but no appreciable changes were observed (Supplementary Figure S3).

To further understand the effects of alisertib on gene expression, we conducted RNA-Seq on a H3K27M DMG patient-derived cell line (DIPGXVII) over a series of time points following treatment with alisertib and analyzed for abundance of AK-related genes. Not surprisingly, the most significant changes occurred in genes related to the cell cycle and mitosis 24–48 hours following alisertib treatment (Figure 2F). To corroborate these findings, we assessed changes in H3K27me3 at similar timepoints following alisertib treatment. The most robust changes equally occurred at the 24- and 48-hour time points (Figure 2G). Together, these data indicate that the cellular changes ­resulting from alisertib-induced AK inhibition are not immediate but rather most prominent 24–48 hours following treatment induction.

### Systemic Delivery of Alisertib Decreases Tumor Burden in Subcutaneous but Not Intracranial H3K27M DMG Models

**Figure 3.**
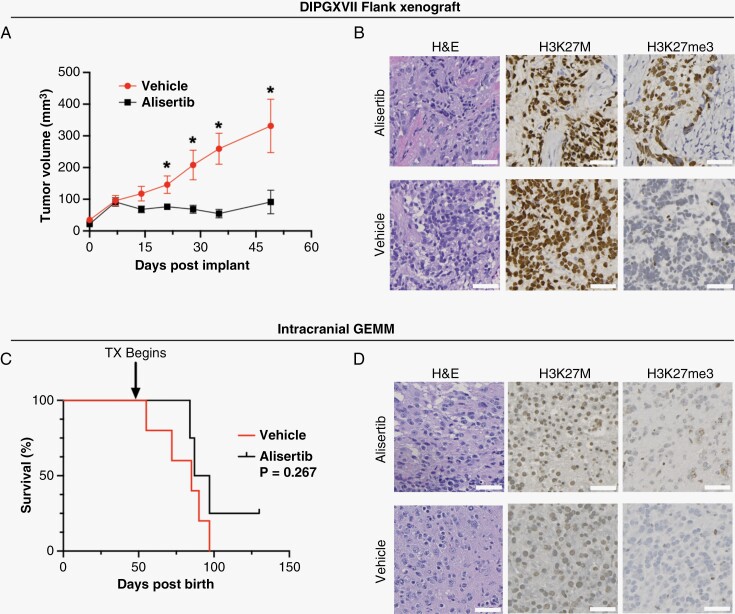
Systemic dosing of alisertib decreases tumor burden in subcutaneous patient-derived xenograft (PDX) but does not improve survival in intracranial H3K27M diffuse midline glioma (DMG) genetically engineered mouse model (GEMM). (A) Tumor volume of H3K27M PDX in flank (DIPGXVII) treated by oral gavage (3x weekly) of alisertib (20mg/kg, *n* = 8) or vehicle (*n* = 8). Differences in volume were assessed with unpaired *t*-test where *P* < .05 was considered significant. (B) Representative histological (H&E) and immunohistochemistry (IHC; H3K27M, H3K27me3) images of formalin-fixed flank tumor sections. Images shown at 20X magnification (scale bars: 50 µm). (C) Kaplan–Meier graph showing percent survival of genetically engineered mouse models (GEMM) dosed daily by oral gavage with alisertib (20mg/kg, *n* = 4) or vehicle (*n* = 5). Differences in survival were determined by Log-Rank Mantel Cox test where *P* < .05 was considered significant. (D) Representative H&E and IHC (H3K27M, H3K27me3) sections of formalin-fixed GEMM brains from animals treated with alisertib or vehicle. Images shown at 20X magnification (scale bars: 50 µm).

As a potent inhibitor that restores part of the epigenetic signature of H3K27M DMG cells in vitro (H3K27me3 increase) and the potential for rapid translation, we proceeded to assess alisertib’s in vivo activity. First, we used a subcutaneous patient-derived xenograft (PDX) model (DIPGXVII) to administer alisertib at 20mg/kg by oral gavage in a daily (7 times/week) dosing regimen. Tumor volume measured by caliper (Figure 3A) and bioluminescence imaging (BLI; Supplementary Figure S4A) significantly decreased in alisertib-treated animals. On-target effects were further observed by immunohistochemistry (IHC), including a robust increase in H3K27me3 and a decrease in H3K27M in alisertib-treated tumors (Figure 3B).

Next, we evaluated the efficacy of the alisertib in 2 intracranial models of H3K27M DMG. We utilized a previously described genetically engineered mouse model (GEMM) that develops spontaneous and highly penetrant brainstem high-grade gliomas with K27M/p53/PDGFα driver mutations following postnatal induction.^10^ We treated MRI-confirmed tumor-bearing GEMM animals with alisertib (20 mg/kg) by daily oral lavage. No changes in tumor burden were observed by MRI (Supplementary Figure S4B), and there was no survival benefit in treated animals (*P* = .267, Figure 3C). Histology (H&E) revealed the presence of tumor cells in both untreated and treated animals, along with no changes in either H3K27M or H3K27me3 upon IHC staining (Figure 3D). Similar results were observed in an intracranial PDX model (DIPGXIIIp*), as there was no difference in tumor size and no survival benefit (*P* = .993) in the treatment group (Supplementary Figure S4C). The results of the flank study indicate that alisertib has the propensity to decrease tumor burden and reverse the epigenetic signature in vivo, but these results were not replicated with intracranial models, suggesting poor brain penetrance of this small molecule drug. This is supported in the literature as Sells et al. and Oh et al. found alisertib to be only approximately 3% brain penetrant.^20^

### CED of Alisertib Does Not Improve Survival in Orthotopic H3K27M DMG Animals

Since systemic delivery of alisertib did not improve survival in multiple intracranial models of H3K27M DMG, we decided to directly administer alisertib using CED. Originally proposed in the 1990s,^21^ CED bypasses the blood-brain barrier (BBB) through a direct, interstitial infusion.^22,23^ By generating a pressure gradient, CED allows for drug delivery directly to the tumor and surrounding tissue while minimizing systemic absorption along with promoting a large, homogenous drug distribution.^24^ CED has been used in adult clinical trials for many years and more recently has been shown safe in children with DMG, giving it exciting clinical application in the treatment of these tumors.^25^

**Figure 4.**
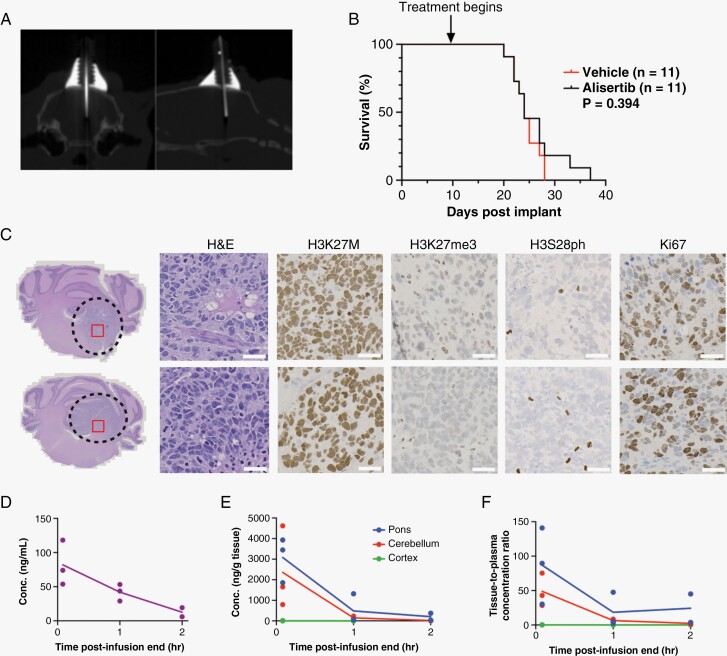
Intracerebral convection-enhanced delivery (CED) of alisertib does not improve survival and is rapidly cleared from an orthotopic H3K27M diffuse midline glioma (DMG) patient-derived xenografts (PDX) model. (A) Computed topography (CT) of brain following CED guide cannula implantation confirms cannula residence in the pons. (B) Kaplan–Meier graph showing percent survival in orthotopic H3K27M DMG PDX (DIPGXIIIp*) treated with CED of alisertib (200 µM, *n* = 11) or vehicle (*n* = 11) twice weekly until moribund. Differences in survival were determined by Log-Rank Mantel Cox test where *P* < .05 was considered significant. (C) Representative H&E of orthotopic H3K27M DMG PDX on day 28 post tumor cell injection at 1X magnification (left). Black dashed line indicates the tumor perimeter, and red square indicates the region from which high-power images were acquired. High-power images (right; H&E, H3K27M, H3K27me3, H3S28ph, and Ki67) shown at 20X magnification (scale bars: 50 µm). (D) Plasma and (E) brain region concentration-time profiles, and (F) tissue-to-plasma concentration ratios following a single CED infusion of alisertib (200 μM) to the pons of non-tumor bearing rats. Data is displayed as individual data points where solid line represents the average. Plasma – Purple, solid purple line; Pons – Blue, solid blue line; Cerebellum – Red, solid red line; Cortex – Green, solid green line.

We used our previously developed CED platform in rat orthotopic PDXs (DIPGXIIIp*).^26^ Ten days following tumor cell injection, a CED guide cannula was implanted, and computed tomography was used to confirm proper cannula placement in the pons (Figure 4A). Animals underwent twice weekly CED infusions of either alisertib (200 μM) or vehicle (experimental workflow depicted in Supplementary Figure S5A). Surprisingly, we did not find a significant difference in overall survival (*P* = .394, Figure 4B). Furthermore, histology and IHC did not reveal specific changes to H&E, H3K27M, H3K27me3, H3S28ph, or Ki67 in alisertib-treated animals compared to controls (Figure 4C).

### Pharmacokinetic Analysis Reveals Short Retention Time of Alisertib in the Brain Following Acute CED

To investigate why CED of alisertib did not prove efficacious, we conducted pharmacokinetic studies to better understand drug disposition in the brain following CED. We performed single CED infusions of alisertib (200 µM) to the pons of non-tumor-bearing rats. Following CED, animals were euthanized at specific time points, and brains were fresh-frozen until they could be dissected into anatomical regions (pons, cerebellum, and cerebral cortex) and analyzed for alisertib content. Concentration-time profiles for plasma (Figure 4D) and brain regions (Figure 4E) as well as brain-to-plasma concentration ratios (Figure 4F) indicated that alisertib is rapidly cleared from the brain following acute CED. The drug retention half-life in the brain was calculated to be 0.493 hours (29.58 minutes) for the pons, 0.281 hours (16.86 minutes) for the cerebellum and 0.12 hours (7.2 minutes) for the cortex. These data indicate that even though we are able to infuse a significant amount of alisertib into the targeted area, the rapid elimination of the drug from the brain could be contributing to its lack of efficacy especially since our in vitro data suggests that alisertib is most effective with 24–48 hours of exposure window.

### Prolonged CED of Alisertib Via Osmotic Pump Improves Survival in Orthotopic H3K27M DMG Animals

**Figure 5.**
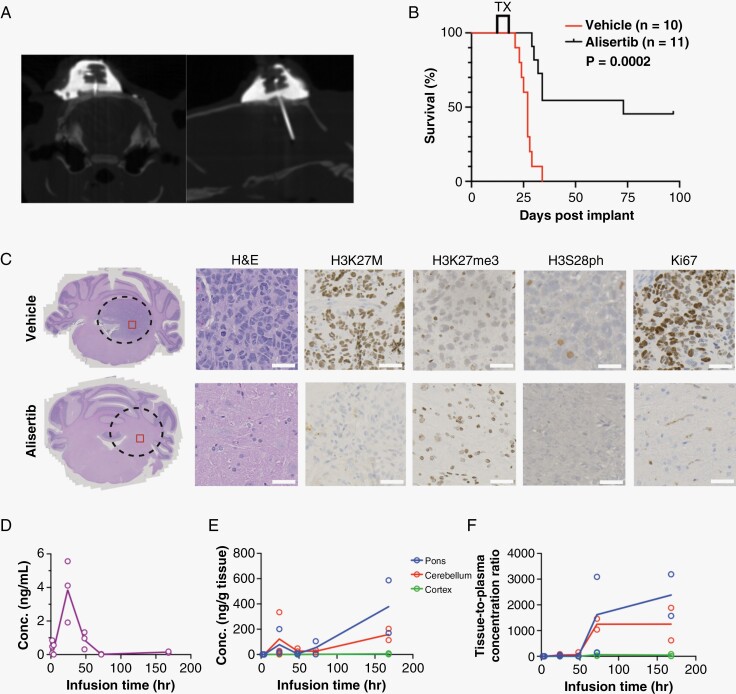
Prolonged convection-enhanced delivery (CED) via an osmotic pump extends survival and improves brain-retention of alisertib in an orthotopic H3K27M diffuse midline glioma (DMG) patient-derived xenografts (PDX) model. (A) computed tomography (CT) brain images following CED cannula and osmotic pump implantation confirm cannula residence in the pons. (B) Kaplan–Meier graph showing percent survival in orthotopic H3K27M DMG PDX (DIPGXIIIp*) treated with 7-day continuous CED of alisertib (200 µM, *n* = 11) or vehicle (*n* = 10). (C) Representative H&E of orthotopic H3K27M DMG PDX either from vehicle-treated or alisertib-treated animals 28 days after injection of tumor cells at 1X magnification (left). Black dashed lines indicate the location of tumor engraftment, and red squares indicate the region from which high-power images were acquired. High-power images (right; H&E, H3K27M, H3K27me3, H3S28ph, and Ki67) shown at 20X magnification (scale bars: 50 µm). (D) Plasma and (E) brain region concentration-time profiles, and (F) tissue-to-plasma concentration ratios following prolonged CED with alisertib (200 μM) to the pons of tumor-naïve rats. Data is displayed as individual data points where solid line represents the average. Plasma – Purple, solid purple line; Pons – Blue, solid blue line; Cerebellum – Red, solid red line; Cortex – Green, solid green line.

In an effort to increase the drug-tumor residence time, we used an alternative CED method, the osmotic ALZET® pump, to continuously deliver alisertib (200 µM) over a course of 7 days. First, we used an orthotopic PDX rat model (DIPGXIIIp*) to allow for a direct comparison with the initial CED study. Animals were implanted with an ALZET® pump and CED cannula 13 days after tumor cell injection (experimental workflow depicted in Supplementary Figure S5B), and computed tomography was used to confirm cannula placement in the pons (Figure 5A). Survival was significantly extended in animals that received alisertib by 7-day continuous CED compared to vehicle controls (*P* = .0002, Figure 5B). There were even a significant portion of long-term survivors that showed no BLI signal and no neurological symptoms over a nearly 100-day period. These animals showed minimal to no tumor cells on H&E while significant burden was visible in the vehicle group. Furthermore, IHC of animals treated with continuous CED showed a decrease in H3K27M, a robust increase in H3K27me3, and decrease of H3S28ph and Ki67 (Figure 5C).

### Pharmacokinetic Analysis Demonstrates Improved Brain Retention of Alisertib With Prolonged CED

To better delineate the CNS kinetics of continuous CED that afforded a significant survival benefit with alisertib in our intracranial PDX model, we again performed pharmacokinetic studies in non-tumor-bearing rats. Animals underwent osmotic ALZET® pump implantation connected to a cannula positioned in the pons. Following the implantation, animals were sacrificed at predetermined time points, and brains were collected and analyzed as previously described. Concentration-time profiles for plasma (Figure 5D) and brain regions (Figure 5E) demonstrated an increase in drug retention in the brain, particularly between 72 and 168 hours (3–7 days). This was further emphasized with brain-to-plasma concentration ratios (Figure 5F), where a high ratio was appreciable in the pons and cerebellum (the targeted regions) even at the end of 7 days of treatment.

### Alisertib Administered Via Prolonged CED Extends Survival in a H3K27M DMG GEMM

**Figure 6.**
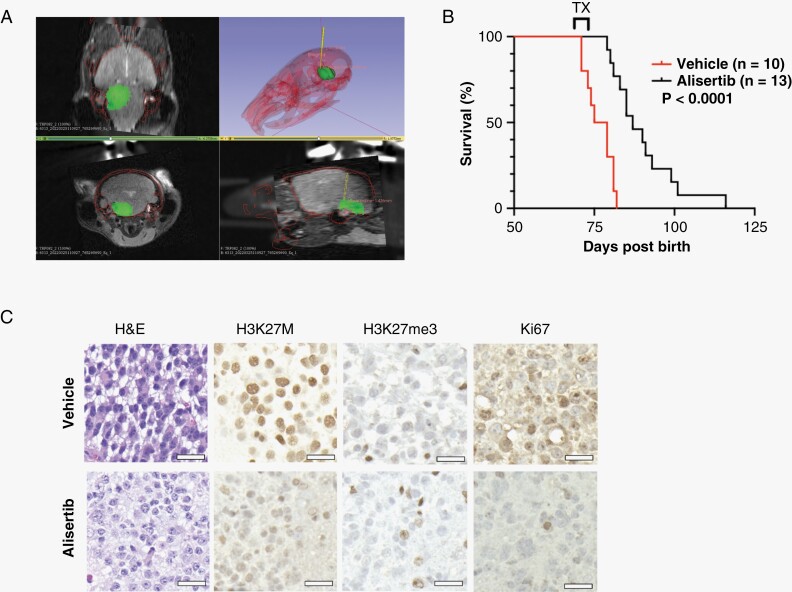
Prolonged convection-enhanced delivery (CED) of alisertib extends survival in a H3K27M diffuse midline glioma (DMG) genetically engineered mouse models (GEMM). (A) Co-registration of CT and magnetic resonance (MR) images into “Stage Space” of 3D Slicer for image-guided CED cannula insertion and 3-dimensional (3D) reconstruction to verify cannula positioning in the targeted tumor (green = tumor, red = skull, and yellow = cannula). Example of tumor segmentation (MRI reconstruction) and probe placement (CT-based alignment) represented in axial slice (bottom), coronal slice (middle), and 3D (top) views. (B) Kaplan–Meier graph showing percent survival in GEMM treated with 7-day continuous CED of alisertib (200µM, *n* = 13) or vehicle (*n* = 10). Differences in survival were determined by Log-Rank Mantel–Cox test where *P* < .05 was considered significant. (C) Representative H&E and IHC (H3K27M, H3K27me3, Ki67) images of formalin-fixed brain sections from vehicle- or alisertib-treated GEMM. Images shown at 40X magnification (scale bars: 20 µm).

To further corroborate our findings in intracranial *H3K27M DMG* PDXs, we chose the previously described GEMM, which has an undisturbed BBB, as no orthotopic tumor cell injection is required. GEMM animals with MRI-confirmed tumors were implanted with ALZET® pumps and CED cannulas on day 70 post-birth. MRI-CT co-registration was performed for planning of stereotactic CED cannula placement and to subsequently confirm proper cannula positioning in the tumor (Figure 6A, Supplementary Table S4). Similar to the orthotopic PDX rat model, alisertib (200µM)-treated mice in the GEMM cohort showed a significant prolongation in survival (*P* < .001, Figure 6B). H&E demonstrated a decrease of tumor cells at the infusion site in alisertib-treated mice while IHC indicated decreased H3K27M, increased H3K27me3, and decreased in Ki67 compared to vehicle-treated animals (Figure 6C). These data suggest that continuous CED of alisertib via an implantable pump is an efficacious treatment strategy against H3K27M DMG. The summation of these data underscores the idea of drug-tumor residence time as a significant barrier for effective treatment of DMG tumors.

## Discussion

Recent landmark studies have provided evidence for the role of epigenetics in H3K27M DMG tumorigenesis and survival. Here, we conducted a cell proliferation- and survival-based drug screen of over 300 epigenetic regulators and identified AK as a target of interest. Inhibitors of AK showed selective and potent antitumor effects, and shRNA knockdowns of both AURKA and AURKB resulted in diminished growth capacity. Opting for an inhibitor with the potential for rapid translation to test in our in vivo models, we found the BBB to prevent a positive effect following systemic administration of alisertib. Initial attempts of loco-regional delivery by CED were also disappointing, and we identified rapid elimination from the target area as an important hurdle for translating promising in vitro results. Increasing drug-tumor residence time with prolonged CED finally resulted in a positive survival benefit in both a GEMM and PDX small animal model.

AKs have been implicated in several types of cancer^27–30^ and previously reported as a potential target for H3K27M DMG tumors.^31^ As a key regulator of mitosis, aberrant AK expression has been affiliated with tumor suppression and oncogenesis.^32–34^ We were able to substantiate the importance of AKs in H3K27M DMG using patient data available from public data bases, patient samples, and with an inducible shRNA knockdown in vitro model. Our in vitro data demonstrate that treatment with AKIs concomitantly increases in H3K27me3 levels, which are classically low in DMG. This supports our hypothesis that inhibitors of this pathway may be an efficacious treatment option for DMG tumors.

Furthermore, AKs are known to phosphorylate H3S10 and H3S28, which leads to chromatin condensation; but how this effects cellular function is not readily known, as other kinases also phosphorylate these residues.^14,35,36^ Our data demonstrate that H3K27M DMG tumors have high levels of H3S10 and H3S28 phosphorylation at baseline, and treatment with AKIs decreased these levels with a concomitant increase in H3K27me3 levels. This data suggests that on-target drug effects, following either H3S10 or H3S28 phosphorylation levels in relationship to drug treatment, could be possible. Intriguingly, H3S28 is immediately adjacent to H3K27, and the reduction in H3K27me3 in these tumors may have an interplay with H3S28 phosphorylation status. Others have shown in 293T cells, H3S28 phosphorylation prevents EZH2 from methylating H3K27,^36^ and there may be a similar mechanism in H3K27M DMG; however, more mechanistic studies will be required to determine the importance of H3S28 phosphorylation^35–37^ in these tumors and is underway in our laboratory.

Specific drug therapy has been one of many hurdles in developing effective treatment for this deadly disease while the BBB is another. The BBB presents a significant anatomical and physiological barrier in drug delivery to brain tumors.^38,39^ The lack of contrast-enhancing features of DMG suggests an intact BBB,^40^ with some evidence that the blood-brainstem barrier is even more difficult to penetrate than the cerebral BBB.^41,42^ Our data supports this theory as we see decreased tumor size and increased K27me3 in our flank model in response to alisertib treatment, where the BBB is not a factor, but do not observe an increase in survival or on-target effects with systemic administration of alisertib in our intracranial GEMM and PDX models.

The difficulties posed by the BBB have instigated research in drug delivery methods focused on circumventing the BBB.^43^ One of these techniques, CED, has been attempted in both adult and pediatric brain tumor populations.^44,45^ Surprisingly, we found that repeat CED of alisertib did not prove efficacious in an orthotopic PDXs. Many variables could contribute to the failure of CED in our animal model and previous clinical trials, such as catheter design, reflux, or volume of distribution, many of which have already been optimized.^24,26,46,47^ One variable that has been neglected until recently is drug elimination from the brain following direct delivery. A recent study by Singleton et al^48^ examined panobinostat elimination from the infusion site following CED, and the authors found a brain retention half-life of only 2.9 hours. Similarly, our data suggest that alisertib resides in the brain for less than one hour following CED, and with such minimal drug-tumor residence time, it is unlikely to achieve treatment efficacy.

Therefore, the new challenge is how to deliver drugs past the BBB and keep them there long enough to successfully impart therapeutic effect. We were able to deliver alisertib directly and continuously with a 7-day osmotic CED pump. This resulted in a significant increase in survival as well as observation of robust on-target drug effects, including several animals who survived long-term. There was a subset of alisertib-treated PDX animals that did not survive long-term but still significantly longer than the vehicle-treated. Even though we have strong data demonstrating the stability of alisertib (Supplementary Figure S6), there are multiple reasons for the observed bimodal response, including a lack of a complete volume of drug distribution to the tumor, or potentially a malfunction with the osmotic pump such as a clogged or possibly dislodged catheter that resulted in the drug to be incompletely delivered.

Our study highlights some of the barriers to successful drug therapy in H3K27M DMG tumors. We used alisertib here because of its potential clinical translation as it was already through phase 2 clinical testing in children (NCT02114229).^49^ Several active studies remain in clinical trial (eg, NCT04555837, NCT04085315, and NCT02114229). One of the benefits of CED for potential future clinical trial efforts is the ability to minimize systemic toxicity through high local concentrations of drug at the target area but relatively low concentrations in the systemic circulation. As opposed to previous studies that investigated the systemic administration of alisertib for targeting CNS tumors,^50^ we did not observe any systemic toxicities in any of our cohorts, which suggests a drug like alisertib would benefit from CED as opposed to systemic delivery.

Our data provide compelling evidence that we need to increase the drug-tumor residence time to translate our preclinical findings to patients. We are in the process of extending these studies to other drugs of interest to validate that this concept is not unique to alisertib. Modulation of drug retention in the target area may include continuous loco-regional delivery using pumps like the one presented in this study. Other mechanisms may be on the horizon as a slow continuous infusion into the brainstem may be challenging in pediatric patients. These include slow-release nanoparticle technology or chemical modifications of drugs to create a “depot” formulation.

Overall, this study suggests that if we are able to (1) select a drug with good antitumor activity and (2) get the drug to cover the tumor area for long enough to achieve the desired effect, then we will finally see positive results in clinical trials—something that is desperately needed.

## Supplementary Material

vdad033_suppl_Supplementary_Material
