# Supplementary material for: Overcoming translational barriers in H3K27-altered diffuse midline glioma: Increasing the drug-tumor residence time
Source: Neurooncol Adv. 2023 Mar 27;5(1):vdad033. doi: 10.1093/noajnl/vdad033 (PMC10148679; doi:10.1093/noajnl/vdad033)
Supplement: vdad033_suppl_Supplementary_Material [file vdad033_suppl_supplementary_material.docx]

**SUPPLEMENTARY METHODS**

*Drug Screen*

A panel of 359 epigenetic regulators were obtained from either Sanford Burnham (261 total drugs) or Nanocyn (98 total drugs). Drugs were dissolved in 100% dimethylsulfoxide (DMSO, Sigma-Aldrich) and stored in 10mM stock. Cells were plated with proper culture media in a 96-well clear bottom black microplate (Corning Costar) at a density of 5 x 10^3^ cells per well and incubated overnight at 37°C and 5% CO_2_. The following day, cells were treated with vehicle (0.5% DMSO) or one of three drug concentrations (0.1μM, 1μM or 10μM in 0.5% DMSO) in triplicate for 72h. Cell viability was assessed using the CellTiter-Blue Cell Viability Assay (Promega) according to the manufacturer’s instructions and an Infinite M200 Pro microplate reader (Tecan). The potency (IC_50_) for each drug was determined using nonlinear least-squares curve-fitting in Prism 9 (GraphPad). Top-hit drugs were further assessed using a 13-point concentration range with 2-fold dilutions.

*Western Blot*

Protein lysates from patient samples were obtained from the Mayo Clinic and the DIPG Registry and Repository (Cincinnati Children’s Hospital Medical Center). In all cases, proper IRB approval and consent was obtained. Tissue sections scraped from fresh frozen slides were lysed in Triton X-100 lysis buffer containing protease inhibitors and sonicated. Protein was quantified with the Pierce Bradford protein assay kit (ThermoFisher Scientific). Then, 10-15μg of total protein was size fractioned by 12.5% SDS-PAGE and transferred to a polyvinylidene difluoride (PVDF) membrane. Specific details regarding antibodies can be found in Supplementary Table 3.

*RNA-Sequencing Analysis*

Quality control was ensured using FASTQC (www.bioinformatics.babraham.ac.uk/projects/fastqc/) and reads were trimmed using a fastx trimmer (hannonlab.cshl.edu/fastx_toolkit/). We mapped trimmed reads to hg19 (patch 13) (genome.ucsc.edu/ENCODE/releaseLog.html) using RNA STAR v2.5 and counted using HTSeq. Then, using edgeR (v3.32.1), differential gene expression analysis and batch normalization was performed with a false discovery rate (FDR) cutoff of <0.05 defining differential expression between conditions. Gene Ontology (geneontology.org) and Kyoto Encyclopedia of Genes and Genomes (KEGG; www.genome.jp/kegg/pathway.html) analysis was used to perform pathway enrichment. Gene Set Enrichment Analyses (GSEA) were performed on normalized counts files where only genes with at least 10 total normalized counts across all replicates were used in order to exclude erroneous gene counts.

**Animal Studies**

*Flank Patient-Derived Xenograft (PDX) Mouse Model*

DIPGXVII cells were transfected with a lentiviral vector pRRLSIN-18.PPT.LUC-GFP.pre (gifted by Dr. Michelle Monje, Stanford University) as previously described ^12^. Cultured cells were resuspended in a single-cell suspension of 1:1 Matrigel (Corning): sterile PBS (Gibco) solution at 10 x 10^5^ cells/μL. Female Hsd:athymic Nude Foxn1nu mice (age 6-7 week old, Charles River Laboratories) received a subcutaneous injection of 2 x 10^6^ cells (200μL) to the flank. Animals underwent systemic drug treatment detailed below.

*Genetically Engineered Mouse Model (GEMM)*

Dr. Suzanne Baker (St. Jude Children’s Hospital) gifted us the spontaneous brainstem glioma mouse model developed in her laboratory ^10^. We bred the respective engineered mice harboring H3f3a^LSL-K27M-Tag/+^,Trp53^flox^ or LSL-PDGFRA^V544ins^ to tamoxifen-inducible Nestin-CreER^T2^ mice. After induction with tamoxifen at P0 and P1, this model results in spontaneous and highly penetrant brainstem high-grade gliomas from postnatal neural progenitors. On postnatal days 50 and 70, animals underwent systemic (oral gavage) and loco-regional (continuous CED) drug treatment, respectively, as detailed below.

*Intracranial PDX Mouse Model*

DIPGXIIIp* cells were transfected with a lentiviral vector pRRLSIN-18.PPT.LUC-GFP.pre (gifted by Dr. Michelle Monje, Stanford University) as previously described ^12^. Cultured cells were resuspended in a single-cell suspension of 1:1 Matrigel (Corning): sterile PBS (Gibco) solution at 1 x 10^5^ cells/μL. Female Hsd:athymic Nude Foxn1nu mice (age 6 – 7 week old, Charles River Laboratories) were anesthetized, a midline incision was made to expose the skull, and a burr hole was drilled using a Foredom MH-130 portable drill (Foredom) 1mm posterior to lambda and 1mm lateral to the mid-sagittal plane. The animal was placed in a stereotactic frame (World Precision Instruments, Model 502650) and 200,000 cells (2μL) DIPGXIIIp* cells were injected into the pons (depth of 4.2mm below the skull) using a 26-gauge 10μL Hamilton syringe at a rate of 0.5μL/min with a Stoelting Quintessential Stereotaxic Injection (QSI). Following the injection, the burr hole was closed with bone wax (Surgical Specialties), and the skin was closed with wound clips (Fine Science Tools).

*Intracranial PDX Rat Model*

DIPGXIIIp* cells were transfected with a lentiviral vector pRRLSIN-18.PPT.LUC-GFP.pre (gifted by Dr. Michelle Monje, Stanford University) as previously described ^12^. On the day of injection, cells were resuspended in sterile PBS (100,000 cells/μL). RHU nude rats (age 5 – 6 weeks; Charles River Laboratories) were anesthetized, a midline incision was made to expose the skull, and a burr hole was drilled using a Foredom MH-130 portable drill (Foredom) 1mm posterior to lambda and 1mm lateral to the mid-sagittal plane. The animal was placed in a stereotactic frame (World Precision Instruments, Model 502650) and 200,000 cells (2μL) DIPGXIIIp* cells were injected into the pons (depth of 7mm below the skull) using a 26-gauge 10μL Hamilton syringe at a rate of 0.5μL/min with a Stoelting Quintessential Stereotaxic Injection (QSI). Following the injection, the burr hole was closed with bone wax (Surgical Specialties), and the skin was closed with wound clips (Fine Science Tools).

*Systemic Drug Treatment of Flank PDX, intracranial PDX, and GEMM*

Animals with a bioluminescence-confirmed flank tumor or MRI-confirmed intracranial tumor were randomized to the control or treatment groups. Alisertib (SelleckChem) was dissolved in 5% DMSO, 50% of 20% cyclodextrin (w/v) and 45% of 2% sodium bicarbonate (w/v) and was administered daily (7 times/week) by oral gavage following 4 -6 hours of fasting prior to gavage. Animals were monitored daily and euthanized at indication of progressive neurological deficit or moribund.

*CED*

1. *Guide Cannula Surgery*

PK STUDY - Female Sprague-Dawley rats (Charles River Laboratories; age 5-6 weeks) were anesthetized, and a midline incision was made to expose the skull. A burr hole was drilled as described above.

EFFICACY STUDY - Animals that underwent successful tumor engraftment were implanted with a CED guide cannula 7 – 8 days post tumor inoculation, where the prior skin incision was reopened to expose the skull.

ALL ANIMALS - Dental glue primer (Kulzer) was applied to the skull surrounding the burr hole with a 0.3mL insulin syringe (BD syringes) and was activated with UV light. The animal was placed in a stereotactic frame, and a stereotactic arm was used to insert a guide cannula (Plastics One, 6mm below the pedestal). Dental glue (Ivoclar Vivadent) was used to affix the guide cannula to the skull. A 26-guage dummy cannula (Plastics One) was inserted into the guide cannula until time of infusion. The incision was closed with wound clips.

1. *Infusion Protocol*

Animals were anesthetized and placed in a stereotactic head frame. The dummy cannula was removed and replaced with a 33-guage internal cannula (Plastics One, 7mm below pedestal) connected via a vinyl catheter to a 500mL Hamilton syringe where the infusion was controlled by a Stoelting QSI. The pump was configured with an infusion rate of 1.0μL/min and a total volume infused of 60μL for the PK studies and 0.5μL/hr and a total volume infused per infusion of 30μL for the efficacy studies. The drug concentration administered was 200μM Alisertib. Animals in the PK study received a single infusion while animals in the efficacy study received twice weekly infusions until neurological deficits/decline was observed or the animals were moribund.

*Alzet*® *Pump Delivery – Rats*

1. *Implantation Surgery*

ALZET PUMP PREPARATION - ALZET® osmotic pumps (Model 2ML1; infusion rate: 10μL/hr) were primed per the manufacturer’s instructions. Each pump received a solution of 200μM alisertib dissolved in 1% DMSO and sterile PBS. The pump was connected to a catheter primed with the drug solution and connected to a 30-gauge cannula (7 mm projection below the pedestal).

PK STUDY - Female Sprague-Dawley rats (Charles River Laboratories; age 5-6 weeks) were anesthetized and a midline incision was made to expose the skull. A burr hole was drilled as previously described.

EFFICACY STUDY - Tumor-bearing animals underwent Alzet pump implantation surgery on day 13 post engraftment. Animals were anesthetized and the previous incision was reopened to expose the skull.

ALL ANIMALS – A subcutaneous pocket was created between the scapulae using a hemostat at the posterior end of the incision. The ALZET® pump connected to the infusion cannula via a vinyl catheter was inserted into the subcutaneous pocket. Dental glue primer (Kulzer) was applied to the skull surrounding the burr hole with a 0.3mL insulin syringe (BD), and the primer was activated with UV light. The animal was placed in a stereotactic frame and a stereotactic insertion arm was used to insert the ALZET® cannula (Plastics One, 7mm below the pedestal). Dental glue (Ivoclar Vivadent) was used to affix the guide cannula to the skull. The skin incision was closed with wound clips.

1. *Pump Removal Procedure*

Animals in the efficacy study who received an ALZET® pump underwent pump removal 8 days post pump implantation (Day 21 post tumor engraftment). Animals were anesthetized and a small incision was made between the scapulae near the pump. The catheter was disconnected and sealed with bone wax, and the pump was removed. The incision was closed with 4.0 vicryl suture (Ethicon).

*Alzet*® *Pump Delivery – GEMM*

1. *Implantation Surgery*

ALZET PUMP PREPARATION - ALZET® osmotic pumps (Model 2001; infusion rate: 1μL/hr) were primed per the manufacturer’s instructions. Each pump received a solution of 200μM alisertib dissolved in 1% DMSO and sterile PBS. The pump was connected to a catheter primed with the drug solution and connected to a 30-gauge cannula (5 mm projection below the pedestal). Animals with MRI confirmed tumors underwent Alzet pump implantation surgery on day 70 post tumor engraftment. Animals were anesthetized and a midline incision was made to expose the skull. A burr hole was drilled using a Foredom MH-130 portable drill (Foredom) at the coordinates determined by MRI for each tumor (Supplementary Table 4). Then, a subcutaneous pocket was created between the scapulae using a hemostat at the posterior end of the incision. The ALZET® pump connected to the infusion cannula via a vinyl catheter was inserted into the subcutaneous pocket. Dental glue primer (Kulzer) was applied to the skull surrounding the burr hole with a 0.3mL insulin syringe (BD), and the primer was activated with UV light. The animal was placed in a stereotactic frame and a stereotactic insertion arm was used to insert the ALZET® cannula (Plastics One, 7mm below the pedestal). Dental glue (Ivoclar Vivadent) was used to affix the guide cannula to the skull. The skin incision was closed with wound clips.

*Imaging*

PDX animals underwent weekly bioluminescent imaging, while intracranial GEMM mice underwent weekly MRI as markers of tumor growth and CT to confirm cannula placement. PDX rats underwent CT scans to confirm accurate cannula placement.

1. *Bioluminescent Imaging (BLI)*

Mice and rats were anesthetized with 2% isoflurane and underwent an intraperitoneal injection of 10mg/kg Cycluc1 (Glixx Laboratories; Hopkinton, MA). Bioluminescence was acquired as previously described ^11^.

1. *Computed Topography (CT)*

Animals anesthetized with 2% isoflurane for the entirety of the scan. Cone-beam CT (CBCT) scans were obtained using a Precision X-RAD SmART rodent irradiator (Precision X-ray, North Bradford, CT) following targeting and alignment in the XYZ planes. A high resolution CBCT protocol (40 kVp, 8.0 mA, 2.0 mm Al filter, 256 images, 100 μm3 voxel size) was utilized for all animals.

1. *Magnetic Resonance Imaging (MRI)*

MR imaging was obtained as previously described ^11^. Briefly, animals were anesthetized with 2% isoflurane and placed in the MRI scanner outfitted with a constant isoflurane inhalation system. MRI was performed using a Bruker Avance 300 MHz (7 Tesla) vertical bore NMR spectroscopy (Bruker Biospin, Billerica, MA). Three-dimensional (3D) T2-weighted fast spin echo sequences were acquired with echo time (TE)/repetition time (TR) = 45.20/2000 ms, bandwidth (BW) = 110 kHz. The total imaging time per mouse was approximately 10 min per scan. Animal core temperatures were maintained at 37 degrees Celsius by a warm air flow.

1. *Co-registration of CT and MRI scans, tumor segmentation, and CED cannula placement validation*

Scans were analyzed using 3D Slicer (www.slicer.org). *CT images were converted into NIfTI format and manually co-registered with all animal-specific MRI scans such that the skull volume visible in the CT scan accurately encompassed the brain volume visualized in the MRI. Tumor dimensions and volume were then defined within the MRI dataset by using the “Segment Editor” module within 3D Slicer to highlight and select all tumor-containing voxels. In this manner, a volumetric reconstruction of the tumor was obtained. CED cannula placement was verified by loading a 3D model catheter probe and aligning in the “Transforms” module to the CED cannula*

*as visualized on post-operative CT scans. Finally, the tumor volume was determined and catheter placement analyses were performed via the “Segment Statistics” module within 3D Slicer.*

*Immunohistochemistry (IHC)*

Samples were dewaxed in xylene and rehydrated with ethanol. Antigen retrieval was performed by steaming slides in a citrate buffer (10mM tri-sodium citrate, 0.05% Tween 20, pH 6.0). Sections were blocked with 10% normal goat serum (NGS) in 1X Tris-buffered saline (TBS) for 30 minutes at room temperature. Primary antibodies were diluted in TBS and 2% NGS and 0.5% Triton X-100 (dilutions listed in Supplementary Table 3). A VECTASAIN Elite ABC kit (VECTOR Laboratory) with a biotinylated secondary antibody was applied per the manufacturer’s instruction and slides were developed with SignalStain DAB Substrate Kit (Cell Signaling) for visualization. Sections were counterstained with hematoxylin and mounted with permount (Fisher Scientific). Images were acquired using a digital slide scanner (Axio Scan.Z1, Zeiss).

*Plasma & Brain Drug Concentration by LC-MS/MS*

Brain samples were homogenized with two tissue volumes of 5% bovine serum albumin (w/v) solution using a homongenizer (PowerGen 125; Thermo Fisher Scientific). A liquid-liquid extraction was performed using 50 μL of each plasma and brain sample, 10 μL of internal standard (500 ng/mL MLN8054), and 500 μL of ethyl acetate. The mixture was vortexed for 5 minutes followed by centrifugation at 14000 rpm at 4°C for five minutes and then, the organic layer was dried under nitrogen. The dried residue was reconstituted in 50 μL of the mixture of 70:30 acetonitrile and distilled water containing 0.1% formic acid before being centrifuged at 14000 rpm at 4°C for five minutes. Five microliters of the supernatant were injected into the Synergi^TM^ Polar-RP column (75 x 2 mm, 4μm, Phenomenex; Torrance, CA) coupled to Agilent 1200 series HPLC system (Agilent, Santa Clara, CA). A gradient elution was employed using 0.1% formic acid in water and 0.1% formic acid in acetonitrile. The flow rate was 0.5 mL/min. The column effluent was monitored with a TSQ Vantage triple stage quadrupole mass spectrometer (Thermo Finnigan, San Jose, CA) at *m/z* 519.1 > 328.1 and *m/z* 477.1 > 316.0 for alisertib and MLN8054, respectively, in positive ESI mode. The calibration curve was sensitive and linear over the range of 1 – 1000 ng/mL and 0.1 – 500 ng/mL for plasma and brain homogenate, respectively**.**

*Alisertib Stability Assay*

Alisertib was dissolved in 1% DMSO and either dPBS or 10% FBS +dPBS to a final concentration of 200μM. 500μL of 200μM alisertib was added to a 1.5mL microcentrifuge tube (Eppendorf) and placed in a pre-heated 37°C themomixer with agitation set to 500rpm to mimic biological conditions for 1, 2 or 4 days (2 replicates per time point, 3 individual experiments). Two samples (T = 0) per experiment were not agitated but flash frozen and stored at -80°C. At the completion of the reaction, timepoints = 1d, 2d, 4d, samples were flash frozen and stored likewise. All samples were stored at -80°C for at least 24 hours prior to HPLC analysis.

For analysis, samples were allowed to thaw at room temperature and then briefly vortexed for 10s. A liquid-liquid extraction was performed as 500μL of sample was mixed with 5mL of DCM. The samples were vortexed for 1 minute and then centrifuged for 1 hour at 4.5 x g at 4°C. The organic layer was dried under nitrogren and the vials were stored at -20°C overnight. The following day, the samples were brought to room temperature and reconstituted in 10% DMSO in MeCN. Each sample was vortexed briefly and sonicated at 40°C for 5 minutes. Finally, the samples were filtered using a 0.2μM nylon syringe. 50μL was injected into the Poroshell 120 C18 column (4.6 mm x 100 mm, 2.7μm, Aligent) coupled to 1260 Infinity II Agilent HPLC system (Agilent). A gradient elution was employed using 0.1% formic acid in water and 0.1% formic acid in MeCN. The flow rate was 1.0 mL/min. The column effluent was monitored with a UV-VIS dectector (Agilent) at 280nm. The calibration curve was sensitive and linear over the range of 0.01 – 1000μM.

**SUPPLEMENTARY FIGURES**

**
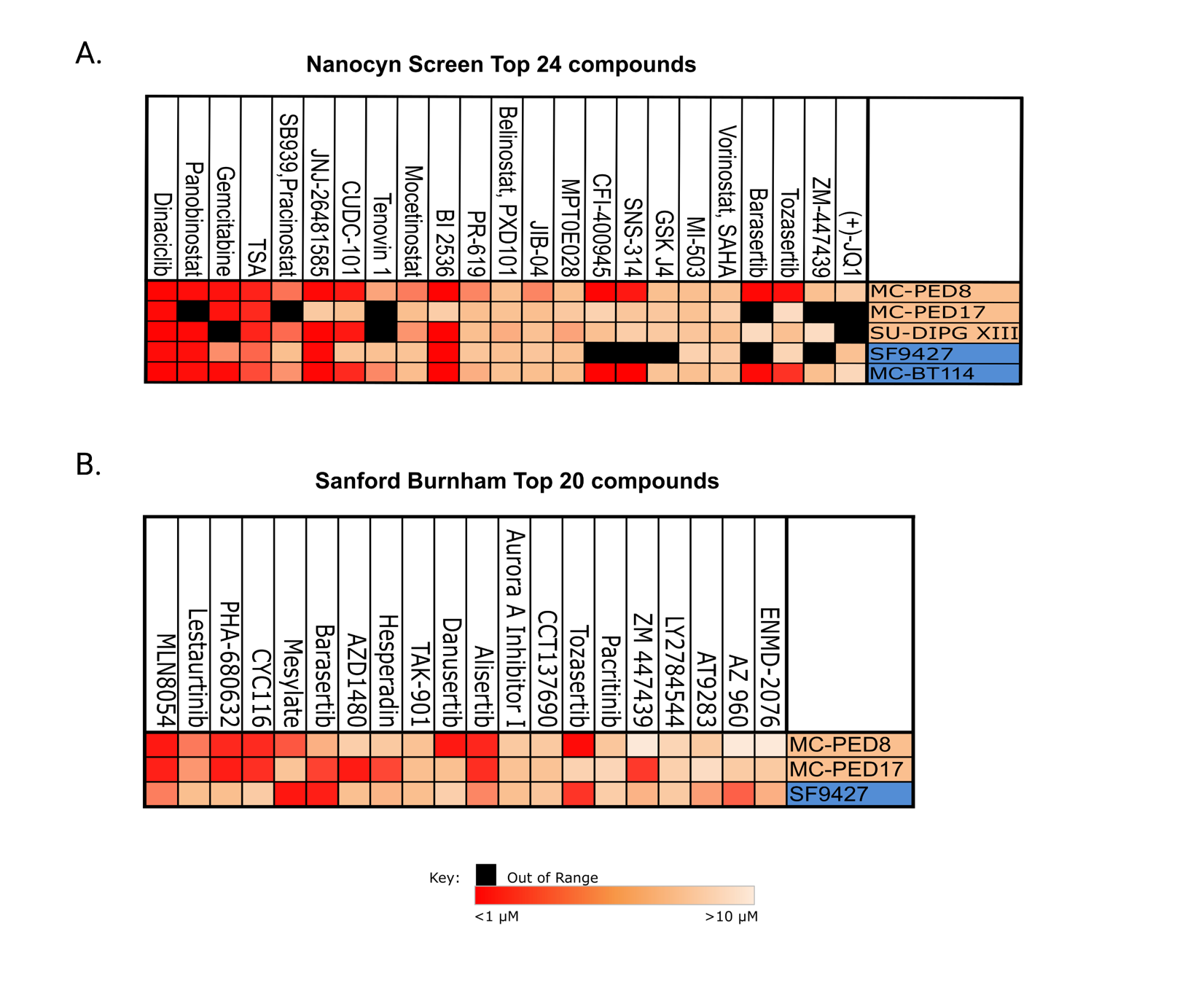
Supplementary Figure 1. Cell viability screen of clinically-relevant epigenetic regulators**. Top compounds from the (A) Nanocyn provided compounds and (B) Sanford Burnham provided compounds. Heatmap intensity is indicative of abosolute IC_50_ with range shown according to the key. Black indicates that the IC_50_ value was outside of measurable range. Each drug was tested in triplicate with 2-3 independent experiments (n = 6-9 per cell line). A complete record of all drugs tested can be viewed in the database provided.

**
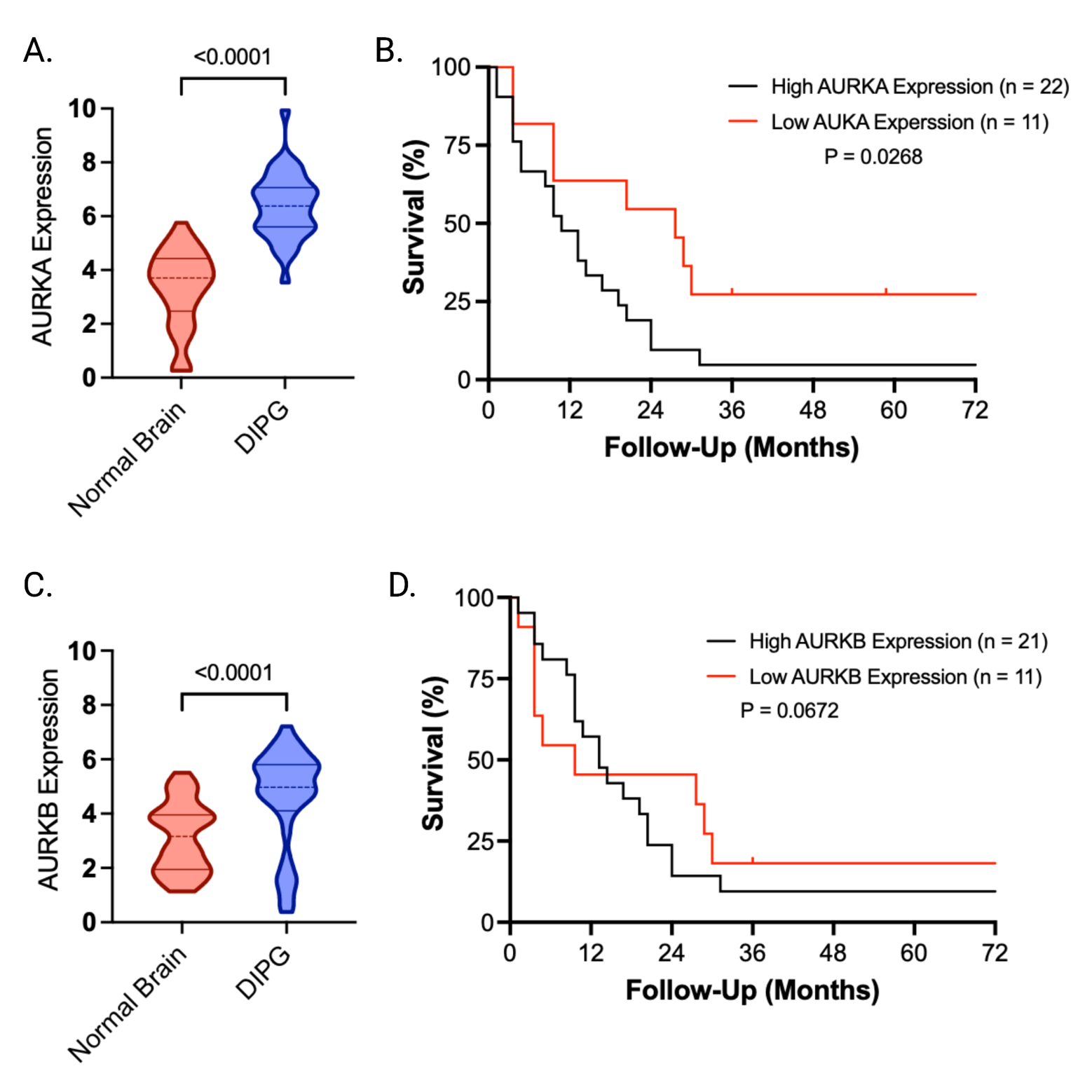
**

**Supplementary Figure 2. Aurora kinase is overexpressed in DIPG patients and associated with a poorer prognosis.** Clinical data accessed from R2: Genomics Analysis and Visualization Platform indicates (A) overexpression of AURKA in DIPG patients (n = 37) compared to normal brain controls (n = 44) and (B) that patients with higher levels of AURKA have poorer prognosis. The same dataset also shows (C) overexpression of AURKB compared to normal brain controls, but (D) without a statistically significant difference in patient survival per their AURKB expression levels. Differences in expression were assessed with a student’s T-test and survival analysis was done using a log-rank Mantel-Cox test. P < 0.05 was considered significant.

**
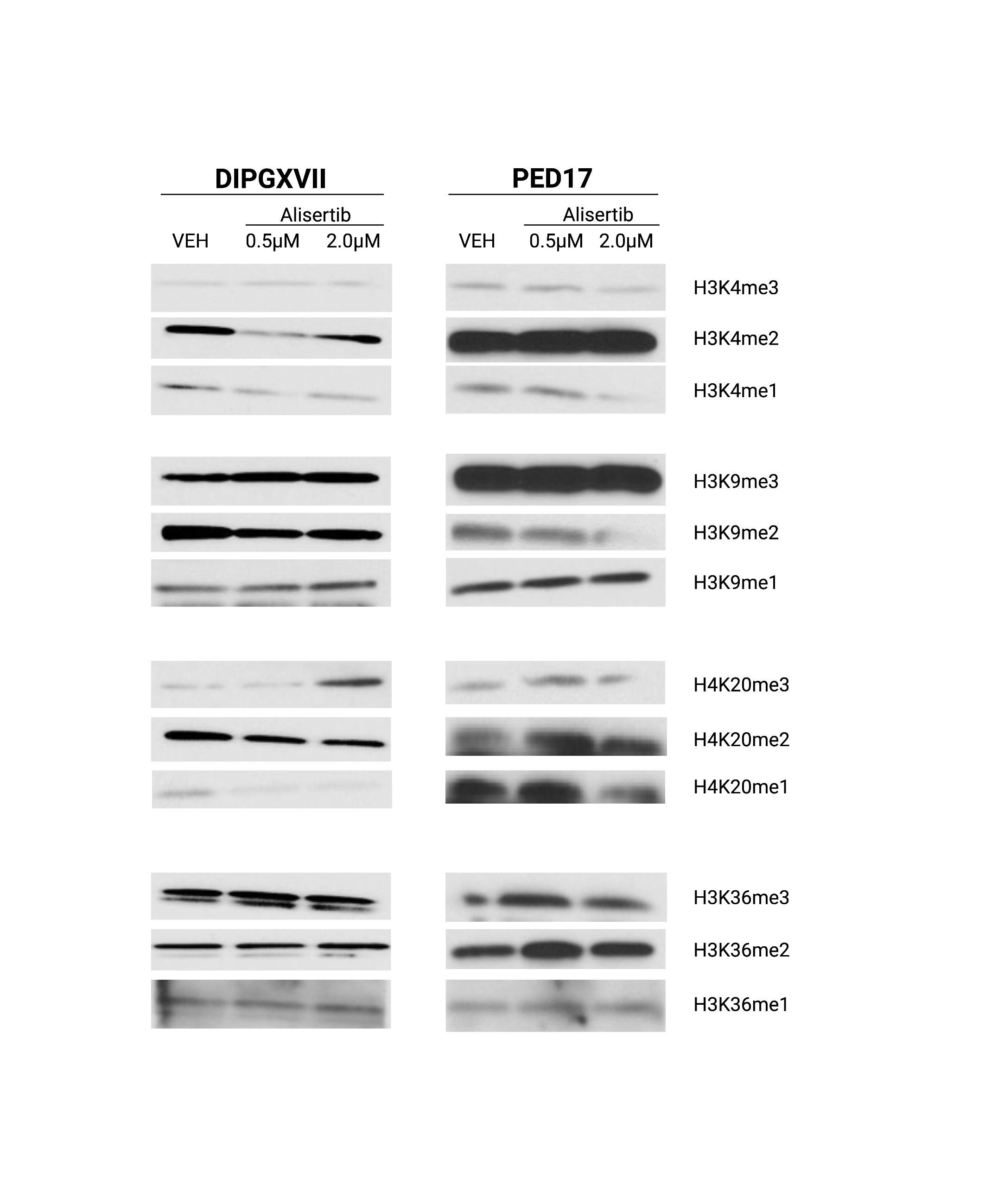
**

**Supplementary Figure 3. Histone H3 and H4 lysine methylation changes resulting from alisertib treatment.** H3K27M patient-derived cell lines (DIPGXVII and PED17) were treated with 0.5μM or 2.0μM alisertib. Mono-, di- and tri-methylation of H3K4, H3K9, H4K20 and H3K36 were assessed by western blot as indicated.

**
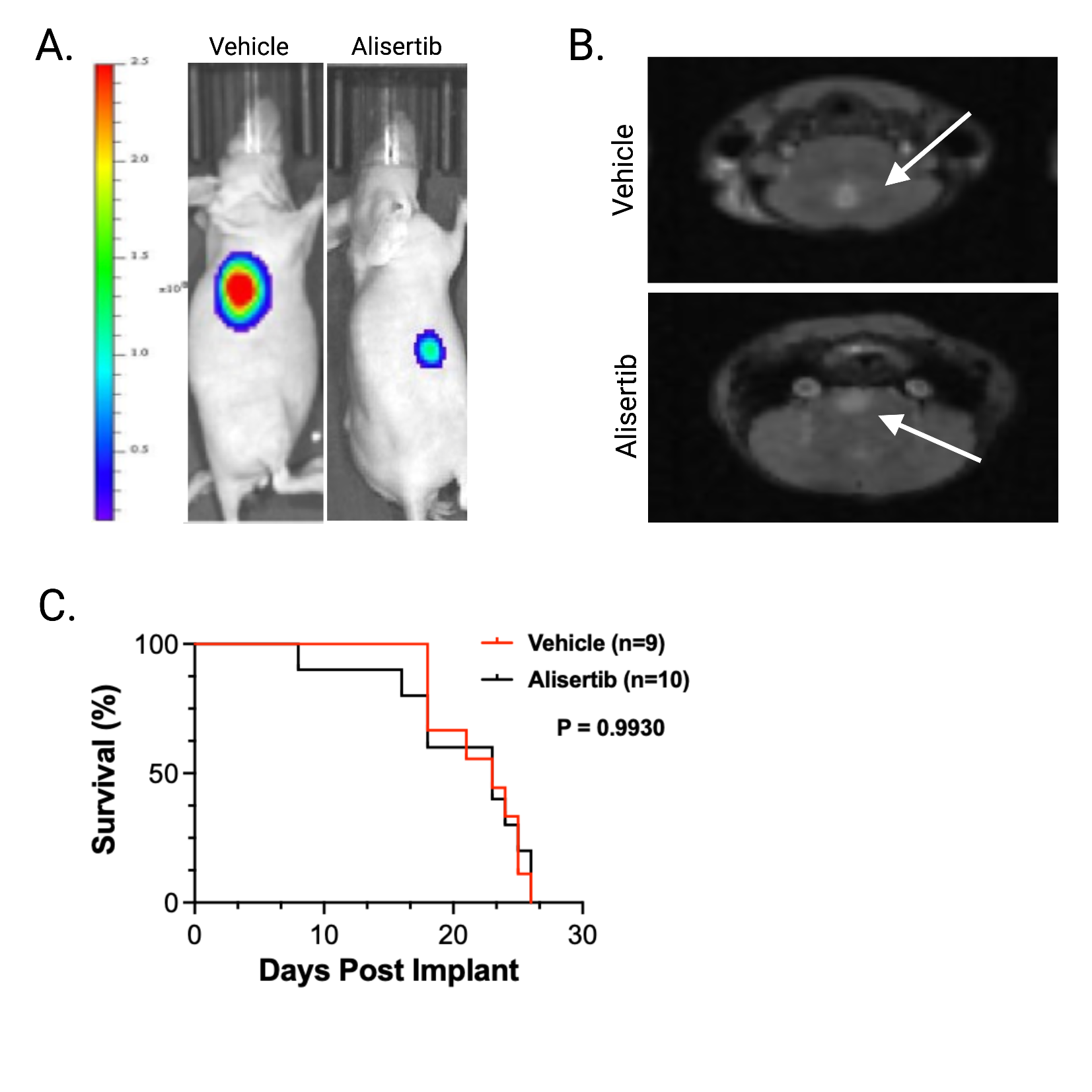
**

**Supplementary Figure 4. Representative Imaging of subcutaneous and intracranial H3K27M tumors following systemic treatment of alisertib.** (A) BLI of DIPGXVII flank patient-derived xenograft (PDX). (B) Axial T2 magnetic resonance imaging (MRI) scan of genetically engineered mouse model (GEMM). (C) Kaplan-Meier graph showing percent survival in DIPGXIIIp* orthotopic PDX with systemic administration (oral gavage, daily) of alisertib (20mg/kg, n=11) or vehicle (n=11). Differences in survival were determined by Log-Rank Mantel Cox test where p<0.05 was considered significant.


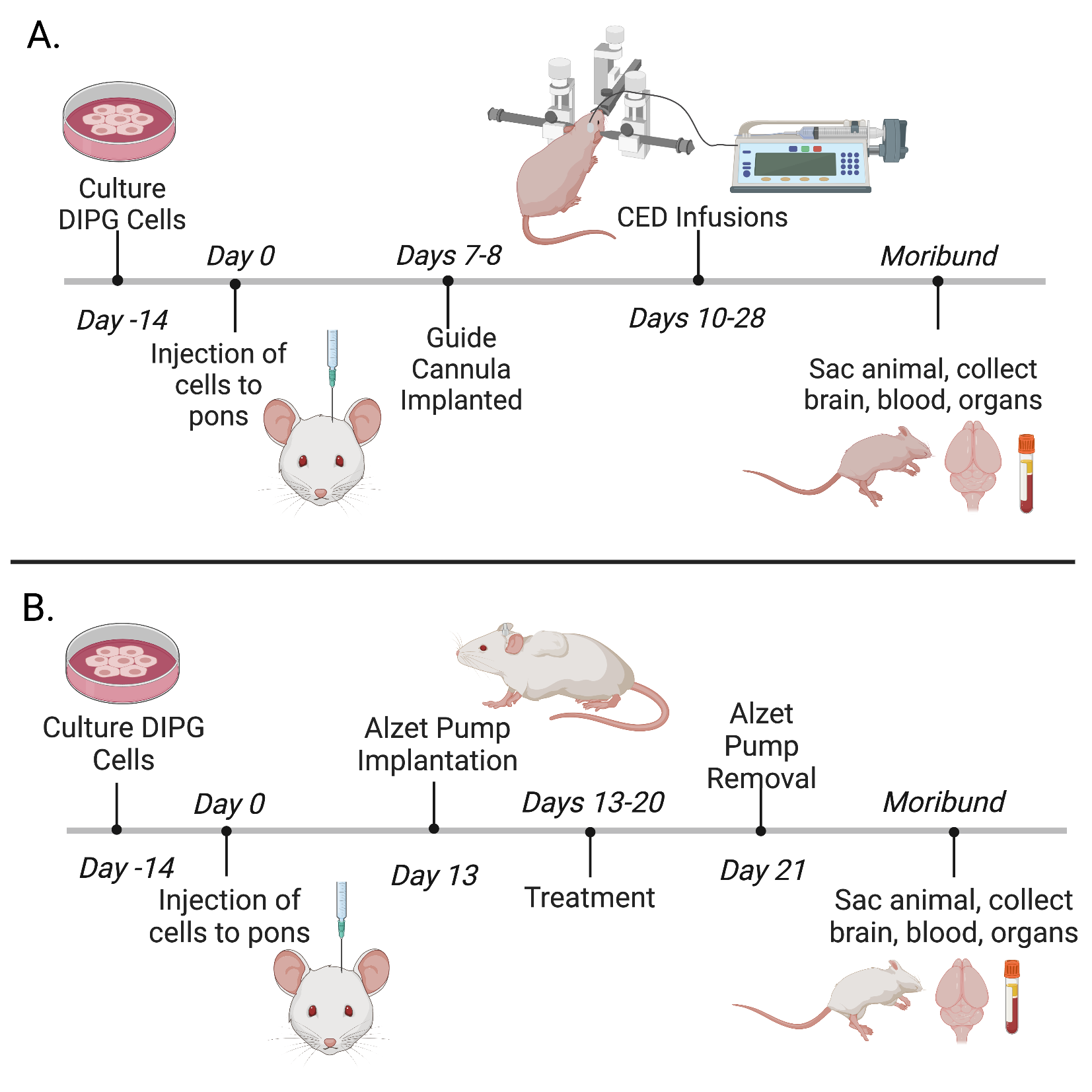


**Supplemental Figure 5.** Experimental schema for DIPGXIIIp* orthotopic PDX rat model which underwent alisertib or vehicle treatment via (A) twice-weekly CED or (B) 7-day continuous CED using an osmotic pump.

**
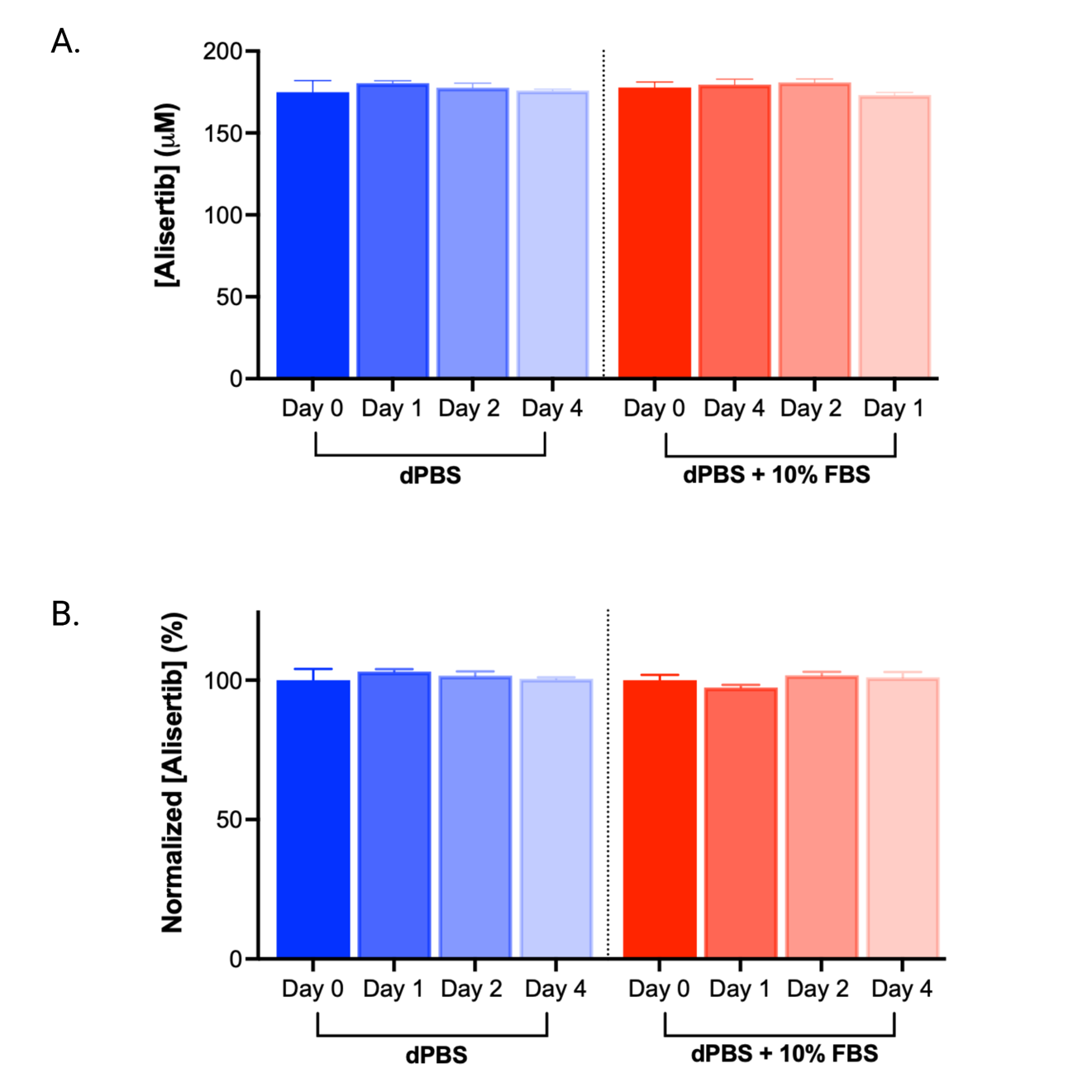
**

**Supplementary Figure 6. Alisertib is stable in solution over time.** Alisertib was dissolved in biologically relevant media (dPBS or dPBS +10% FBS) to a final concentration of 200μM at 1% DMSO. Samples were continuously agitated at 37°C at timepoints indicated and then analyzed using HPLC to determine alisertib concentration (A). Samples were normalized to day 0 samples (B) further demonstrating stability.

**SUPPLEMENTARY TABLES**

**Supplementary Table 1**. Details regarding demographic, molecular, historical and cell culture data on the cell lines used in this study*.

| Cell Line ID | Age/Sex | Institution | Molecular Status | Tests | Date of Tissue Collection | Culture Media |
| --- | --- | --- | --- | --- | --- | --- |
| BT114 | M | Mayo Clinic | GBM, IDH WT | Sanger, STR | 2009 | MHM |
| PED8 | 14/M | Mayo Clinic | H3.3-K27M | Sanger, STR | 2014 | MHM |
| PED17 | 12/F | Mayo Clinic | H3.3-K27M | Sanger, STR | 2015 | MHM |
| SU-DIPGXIIIp* | 6/F | Stanford University | H3.3-K27M | Sanger, STR | Received 2016 | TSM |
| SU-DIPGXVII | 8/M | Stanford University | H3.3-K27M | Sanger, STR | Received 2016 | MHM |
| SF8628 | 3/F | Millipore/Hormel Institute | H3.3-K27M | Sanger, STR | Purchased 2019 | dBT |
| SF9427 | 9/F | UCSF | GBM, IDH WT | Sanger, STR | Received 2018 | N5 |

*All H3K27M cell lines were validated for H3K27M expression by Western blot and Sanger sequencing every three months, and all cell lines were validated by short tandem repeat (STR) DNA fingerprinting annually as well as mycoplasma testing every three months.

Abbreviations: GBM – Glioblastoma; IDH – Isocitrate Dehydrogenase; WT – Wildtype; STR – Short

Tandem Repeat; UCSF – University of California San Francisco.

**Supplementary Table 2.** Media composition for culture of patient-derived cell lines.

| Cell Line | Media Name | Media Contents |
| --- | --- | --- |
| BT114, PED8, PED17, SU-DIPGXVII | Serum-free complete media (MHM) | DMEM/F12 (HyClone), 25 mM glucose (Sigma-Aldrich), 8.9 mM sodium bicarbonate (Life Technologies), 2 mM glutamine (Life Technologies), 4 mM HEPES (Life Technologies), 1% penicillin/streptomycin (Life Technologies), N2 supplement (Life Technologies), 4 μg/mL heparin (Sigma Aldrich), 20 ng/mL human EGF (PeproTech US), 20 ng/mL human b-FGF (PeproTech US), 20 ng/mL human PDGF AA with 20 ng/mL human PDGF BB (Shenandoah Biotechnology). |
| SU-DIPGXIII, SU-DIPGXIIIp* | Tumor stem cell medium (TSM) | Neurobasal (-A) (Life Technologies), DMEM/F12 (Life Technologies), B27 (-A) (Life Technologies), 4 μg.mL heparin (Sigma Aldrich), 20 ng/mL human EGF (PeproTech US), 20 ng/mL human b-FGF (PeproTech US), 20 ng/mL human PDGF AA with 20 ng/mL human PDGF BB (Shenandoah Biotechnology). |
| SF8628 | dBT medium | DMEM/F12 (Hyclone), 10% FBS |
| SF9427 | N5 medium | TSM medium supplemented with 5% FBS (Atlanta Biologicals) |

**Supplementary Table 3.** Antibody dilutions used for Western Blot (WB) and immunohistochemistry (IHC).

| Antibodies | Source | Identifier | Dilution for WB | Dilution for IHC |
| --- | --- | --- | --- | --- |
| H3K27M | Abcam | Ab190631 | 1: 10,000 | 1:400 |
| AURKA | Cell Signaling | 14475S | 1: 1,000 | - |
| AURKB | Cell Signaling | 3094S | 1: 1,000 | - |
| H3S10ph | Cell Signaling | 9701 | 1: 3,000 | - |
| H3S28ph | Cell Signaling | 9713S | 1: 3,000 | 1:200 |
| phAURKA/B/C | Cell Signaling | 2914 | 1: 250 | - |
| α-Tubulin | Sigma | T9026 | 1:10,000 | - |
| Ki67 | Dako | M7240 |  | 1:500 |
| H3K4me1 | Cell Signaling | 5326 | 1: 2,000 | - |
| H3K4me2 | AbCam | Ab7766 | 1: 3,000 | - |
| H3K4me3 | Millipore | 04-745 | 1: 2,000 | - |
| H3K9me1 | AbCam | Ab8896 | 1: 3,000 | - |
| H3K9me2 | AbCam | Ab1220 | 1: 1,000 | - |
| H3K9me3 | AbCam | Ab8898 | 1: 3,000 | - |
| H3K20me1 | AbCam | AB9051 | 1: 1,000 | - |
| H3K20me2 | Cell Signaling | 9759 | 1: 1,000 | - |
| H3K20me3 | Millipore | 07-463 | 1: 1,000 | - |
| H3K27me1 | Millipore | 07-448 | 1: 3,000 | - |
| H3K27me2 | Cell Signaling | 9728 | 1: 2,000 | - |
| H3K27me3 | Cell Signaling | 9733S | 1: 500 | 1:100 |
| H3K36me1 | Millipore | 07-548 | 1: 500 | - |
| H3K36me2 | Cell Signaling | 2901 | 1: 3,000 | - |
| H3K36me3 | AbCam | Ab9050 | 1: 3,000 | - |

**Supplementary Table 4.** GEMM tumor characteristics and image-guided cannula placement.

| **Parameter** | **Outcome (n=20 animals)*** |
| --- | --- |
| **Primary tumor location** |  |
| Brainstem | 6 |
| Cerebellum | 9 |
| Hemisphere | 5 |
| **Intracranial side of primary tumor** |  |
| Left | 10 |
| Right | 9 |
| Midline | 1 |
| **Mean tumor volume [mm^3^]** |  |
| Total | 9.95 ± 6.55 |
| Brainstem tumors | 10.14 ± 8.33 |
| Cerebellar tumors | 9.47 ± 5.76 |
| Hemispheric tumors | 10.59 ± 7.04 |
| **Cannula tip in tumor** |  |
| Yes | 12 |
| No | 8 |
| **Mean distance cannula tip to tumor center [mm]** |  |
| Total | 1.24 ± 0.61 |
| Brainstem tumors | 1.00 ± 0.49 |
| Cerebellar tumors | 1.39 ± 0.68 |
| Hemispheric tumors | 1.24 ± 0.66 |

*Categorical data reported as n (% total), continuous data reported as mean ± SD.
